# A Comprehensive Review of Sonographic Assessment of Peripheral Slow-Flow Vascular Malformations

**DOI:** 10.7759/cureus.54099

**Published:** 2024-02-12

**Authors:** Sheetal S Shelar, Rajasbala Dhande, Pratap Parihar, Neha D Shetty, Shreya Khandelwal

**Affiliations:** 1 Radiodiagnosis, Jawaharlal Nehru Medical College, Datta Meghe Institute of Higher Education and Research, Wardha, IND

**Keywords:** treatment strategies, multimodal imaging, diagnostic accuracy, vascular anomalies, sonographic assessment, peripheral slow flow vascular malformations

## Abstract

This comprehensive review explores the role of sonographic assessment in diagnosing and characterizing peripheral slow-flow vascular malformations (PSFVM). The review begins with an introduction providing the background and significance of PSFVM, defining these vascular anomalies, and emphasizing the importance of sonography in their diagnosis. The objectives focus on a thorough examination of existing literature, assessing the effectiveness of sonography in delineating morphological and hemodynamic features crucial for accurate classification. The summary of key findings highlights the diagnostic accuracy of sonography while acknowledging its limitations. Implications for clinical practice emphasize the practical utility of sonography in early diagnosis and preoperative planning, suggesting integration into multimodal approaches. The conclusion underscores the need for standardized criteria, ongoing education, and future research, positioning sonography as a valuable tool in the comprehensive management of PSFVM.

## Introduction and background

Peripheral slow-flow vascular malformations (PSFVM) refer to a heterogeneous group of vascular anomalies characterized by abnormal connections between arteries, veins, capillaries, and lymphatic vessels. These malformations exhibit a slow and often unpredictable blood flow, presenting a diagnostic challenge for clinicians. The complexity of these lesions necessitates advanced imaging techniques for accurate diagnosis and characterization [1]. PSFVM involve aberrant vascular structures that result from developmental anomalies in the embryonic vascular system. These anomalies can manifest as venous malformations, lymphatic malformations, capillary malformations, or arteriovenous malformations, contributing to a spectrum of clinical presentations and complications [2]. The significance of sonographic assessment in diagnosing PSFVM lies in its non-invasive nature and ability to provide real-time, dynamic imaging. Sonography is crucial in identifying these malformations' morphological and hemodynamic features, aiding in accurate classification and guiding subsequent treatment decisions. Additionally, sonography is often preferred for its cost-effectiveness and lack of ionizing radiation compared to other imaging modalities [3].

A comprehensive review of the existing literature is essential to understand the evolution of sonographic techniques in diagnosing and characterizing PSFVM. This section will explore the various studies, methodologies, and findings related to sonography in the context of PSFVM, highlighting advancements and areas that require further investigation. This review aims to critically assess sonography's effectiveness as a diagnostic tool for PSFVM. By evaluating the sensitivity and specificity of sonographic criteria, we aim to determine the reliability of this imaging modality in differentiating PSFVM from other vascular lesions and establishing a basis for its clinical utility. This evaluation will also consider the limitations and challenges of sonographic assessment in this context.

## Review

Sonographic techniques for PSFVM assessment

High-Resolution Ultrasound Imaging

Doppler ultrasound: Doppler ultrasound emerges as an invaluable tool in evaluating PSFVM, offering a detailed assessment of the flow and velocity characteristics inherent in these anomalies. Color Doppler imaging is essential in discerning between high- and low-flow lesions. High-flow anomalies, exemplified by arteriovenous malformations (AVMs), are identified through the confirmation of elevated systolic flow and arterialization of veins, a distinction made possible through color Doppler and spectral analysis. Conversely, low-flow lesions, encompassing venous malformations (VMs) and lymphatic malformations, manifest distinctive flow patterns on Doppler imaging, including monophasic or biphasic flow in venous lesions and the absence of flow in lymphatic lesions [4-6]. In PSFVM, Doppler ultrasound proves mainly instrumental in categorizing vascular anomalies into high- or low-flow lesions, laying the foundation for subsequent diagnosis and management. Its efficacy extends to the differentiation between various types of vascular anomalies, such as hemangiomas, AVMs, and VMs, thereby significantly contributing to precise clinic radiological diagnoses in most cases [6,7]. Furthermore, the adaptability of Doppler ultrasound, achieved through adjusting parameters such as pulse repetition frequency, Doppler gain, and wall filter, facilitates the detection of slow velocity, thereby enhancing its utility in the comprehensive assessment of PSFVMs [7].

Color Doppler imaging: High-resolution ultrasound imaging, coupled with color Doppler imaging, is a pivotal technique in the comprehensive assessment of PSFVM. This approach facilitates the categorization of vascular anomalies into high- or low-flow lesions, establishing a crucial foundation for subsequent diagnostic and management decisions. Doppler imaging plays a central role in differentiating between high- and low-flow lesions, encompassing capillary, venous, and lymphatic malformations. Doppler imaging is the first-line imaging modality for diagnosing vascular anomalies, particularly in the pediatric population. It aids in confirming high-systolic flow in high-flow lesions, providing a nuanced differentiation between vascular and lymphatic malformations. High-frequency transducers (12-17 MHz) are recommended for ultrasound examinations, ensuring detailed imaging of these complex anomalies [4,6,7]. The step-by-step Doppler ultrasound approach to diagnosing vascular anomalies involves evaluating the presence or absence of arterial flow. The presence of arterial flow indicates a high-flow lesion, while the absence suggests a low-flow lesion. This systematic approach and clinical findings contribute to precise clinic radiological diagnoses in most cases. While ultrasound remains a cornerstone, imaging modalities such as magnetic resonance imaging (MRI) or computed tomography (CT) are often employed, especially for high-flow lesions. MRI/angiography proves essential in defining the morphology of high-flow lesions. In slow-flow malformations, direct percutaneous phlebography emerges as a valuable tool, offering detailed insights into intralesional flow patterns and potential associations with other anomalies. Overall, various imaging techniques, including color-coded Duplex ultrasound, contrast-enhanced ultrasound (CEUS), 4D computed tomography angiography (CTA), and MRI, play a crucial role in classifying vascular malformations based on their dominant vessel anatomical extension and flow pattern [8].

Power Doppler imaging: High-resolution ultrasound imaging, complemented by power Doppler imaging, emerges as an invaluable technique for the sonographic assessment of PSFVMs [8]. This modality provides a detailed examination, enabling the differentiation between slow-flow and high-flow lesions. Color-coded duplex ultrasound proves informative, revealing sluggish blood flow within dysplastic vessels and aiding in the crucial differentiation between slow-flow and high-flow lesions [6,8]. A systematic, step-by-step sonographic approach to diagnosing vascular anomalies involves assessing the presence or absence of arterial flow. The presence of arterial flow indicates a high-flow lesion, while low-flow lesions exhibit the absence of arterial flow [6]. A comprehensive review of ultrasound evaluation in PSFVMs underscores key findings that significantly contribute to the diagnosis of these conditions [7]. This emphasizes the importance of ultrasound, particularly with power Doppler imaging, as a primary diagnostic tool for evaluating PSFVM, offering valuable insights into their hemodynamics and aiding in precise clinical decision-making.

Contrast-Enhanced Ultrasound (CEUS)

High-resolution ultrasound, incorporating CEUS, is a valuable and versatile technique for comprehensively assessing PSFVMs. Utilizing color-coded Duplex ultrasound, this modality effectively delineates slow blood flow within dysplastic vessels, facilitating the crucial differentiation between slow-flow and high-flow lesions [8]. A systematic, step-by-step sonographic approach involves evaluating the presence or absence of arterial flow, where the presence of arterial flow signifies a high-flow lesion. In contrast, low-flow lesions exhibit an absence of arterial flow [6]. The ultrasound characteristics of PSFVMs have been subject to review, underscoring the pivotal role of ultrasound in evaluating and understanding these complex conditions [7]. High-resolution ultrasound, encompassing advanced techniques like CEUS, is critical in the sonographic assessment of PSFVMs. This approach enables accurate diagnosis and effective classification of these vascular anomalies, emphasizing its importance in guiding appropriate clinical decisions and management strategies.

Other Emerging Sonographic Techniques

4D sonography: Integrating 3D/4D volume transducers into sonography has revolutionized imaging capabilities by providing a real-time, 3D view of patient anatomy [9]. This advancement enables sonographers to visualize structures dynamically, offering enhanced spatial perspectives. The ability to capture movement in real-time is precious in various medical applications, including assessing PSFVMs, where understanding the dynamic nature of blood flow and anatomical structures is crucial for accurate diagnosis.

One-touch imaging: Implementing one-touch imaging represents a significant leap in simplifying the sonographic imaging process. This technique automatically adjusts multiple imaging parameters, such as frequency, focus, and compounding, whenever the depth setting changes [10]. This automation streamlines the workflow for sonographers and ensures consistency and precision in imaging. In the context of PSFVM assessment, where detailed and accurate imaging is paramount, this technology simplifies the operation while maintaining the quality of the diagnostic information.

Automated lesion segmentation: Addressing the challenges of manual lesion measurement, automated lesion segmentation software has emerged as a valuable tool. This technology eliminates manual delineation by automatically segmenting lesions, contributing to improved reproducibility irrespective of the sonographer's experience [10]. In the context of PSFVM diagnosis, automated segmentation ensures standardized measurements, reducing variability and enhancing the reliability of assessments over time.

Artificial intelligence (AI) integration: The increasing integration of AI into ultrasound systems signifies a paradigm shift in imaging technology. AI algorithms are designed to improve image quality, reduce errors, and streamline workflows [10]. In assessing PSFVMs, AI integration can enhance diagnostic accuracy by identifying and classifying vascular anomalies, ultimately contributing to more informed clinical decisions.

Tissue tension measurement: A novel ultrasound method for measuring tissue tension introduces a promising avenue for diagnostic innovation. This technique has the potential to quantify tension levels in human tissue, offering insights into abnormal tissue conditions, scarring, and even cancer [11]. In the context of PSFVMs, where tissue characteristics play a crucial role in diagnosis, this advancement may provide additional diagnostic parameters for a more comprehensive assessment.

Ultrasound-guided techniques: Ultrasound-guided techniques have expanded the scope of ultrasound applications beyond traditional imaging. These techniques find utility in disease modeling and drug administration, presenting a new frontier in ultrasound's role in medical research and therapeutic interventions [12]. In the context of PSFVMs, these guided techniques may offer innovative approaches to studying vascular anomalies and exploring potential targeted treatments.

Diagnostic criteria for PSFVM

Morphological Characteristics

Size and configuration of the lesion: The dimensions and structure of PSFVMs exhibit variability, presenting as subcutaneous lesions of diverse shapes and sizes. A specific subtype, VMs, manifests as globular spongy lesions, potentially causing congestive pain [13]. These malformations may appear as focal bluish lesions that expand under certain conditions, resulting in bleeding and pain when congestion or clotting occurs within the malformation [14]. Accurate diagnostic imaging necessitates the detailed specification of the malformation's exact size, shape, and morphology, which is crucial for a comprehensive assessment. MRI further aids in characterizing these malformations, with VMs revealing distinctive features, such as phleboliths [15].

Internal echogenicity: The ultrasound assessment of PSFVMs, encompassing entities like VMs, involves the examination of internal echogenicity. PSFVM, characterized as low-flow lesions, may exhibit heterogeneous echoes during sonographic evaluation [4]. The sonographic assessment of vascular malformations, including PSFVM, focuses explicitly on appraising the internal echogenicity of the lesions. For instance, congenital fibrosarcomas are identified as solid masses with heterogeneous echogenicity during sonographic evaluation [16]. This evaluation is a pivotal component of the diagnostic process, contributing to the characterization and distinction of these conditions from other soft tissue masses.

Hemodynamic Features

Blood flow patterns: Hemodynamic features, specifically blood flow patterns, are pivotal in classifying peripheral vascular malformations (PVMs). The distinction between high-flow and low-flow malformations is paramount in understanding the pathophysiology of these anomalies. High-flow malformations are characterized by the simultaneous presence of arterial and venous phases within a single angiographic image, often accompanied by arteriovenous shunting. Conversely, low-flow malformations lack the demonstration of arterial and venous phases in the same image and are further categorized into capillary malformations, lymphatic malformations, and VMs [1]. Various imaging modalities, including color Doppler ultrasound, CEUS, 4D CTA, and dynamic MRI, are employed to precisely assess flow dynamics, facilitating the differentiation between high-flow and low-flow PVMs [8,15,17]. The elucidation of these hemodynamic characteristics is essential in ensuring an accurate diagnosis and classification of vascular malformations, thereby guiding clinicians in making informed decisions regarding appropriate management and treatment strategies [1,8,18]. This nuanced understanding of blood flow patterns is a cornerstone in the comprehensive evaluation of PVMs, contributing to improved patient care and outcomes.

Velocity Measurements

Blood flow velocity: The measurement of blood flow velocity can be accomplished through various methods, such as video capillary microscopic with frame-to-frame analysis or laser Doppler anemometry [19]. In the context of PSFVMs, the blood flow velocity tends to be notably slower when compared to that in normal vessels.

Cerebral blood flow (CBF): Assessing cerebral hemodynamics relies heavily on the crucial parameter of CBF. Functional ultrasound imaging, a Doppler-based technique, facilitates real-time CBF measurement [20]. Within PSFVMs, CBF is often reduced when contrasted with the flow in normal vessels.

Cerebral blood volume (CBV): Another pivotal parameter in brain hemodynamics is CBV, which can be accurately measured through functional ultrasound imaging, providing comprehensive insights into both CBF and CBV [20]. In the presence of PSFVMs, alterations in CBV are frequently observed compared to the norm.

Red blood cell velocity (RBCv): The assessment of blood flow in small cortical vessels involves critical parameters, with RBCv being central to this evaluation. While functional ultrasound imaging can be employed to measure RBCv in small vessels, it may pose challenges due to the broad spectrum of the Doppler signal [20]. Within the context of PSFVMs, a reduction in RBCv is commonly identified when contrasted with the flow in normal vessels.

Velocity profiles: Graphically representing velocity fields by plotting velocity as a function of position yields a velocity profile [21]. In PSFVMs, the velocity profile often deviates from that observed in normal vessels, providing valuable diagnostic insights. Healthcare professionals can meticulously assess the hemodynamic features and velocity measurements associated with PSFVMs using these diagnostic criteria and advanced imaging techniques. This nuanced understanding significantly contributes to accurately diagnosing and effectively managing these intricate vascular malformations.

Differential Diagnosis Considerations

The diagnostic criteria for PSFVMs entail a thorough evaluation of hemodynamic features, a vital step in distinguishing these malformations from other vascular conditions. Of particular importance in this assessment is the consideration of blood flow velocity. Color Doppler ultrasound is a prevalent tool in assessing these malformations' flow and velocity characteristics, facilitating the differentiation between slow-flow and high-flow lesions [4]. Furthermore, quantitative hemodynamic measurements, including parameters such as RBCv and blood flow velocity, play a crucial role in elucidating the hemodynamics of vascular malformations [20]. While various methods, such as functional ultrasound imaging, offer precise measurements of hemodynamic features, evaluating blood flow velocity remains a pivotal aspect of the diagnostic criteria for PSFVMs [20]. This comprehensive approach ensures a nuanced understanding of the hemodynamic characteristics, accurately diagnosing PSFVMs.

Clinical applications of sonographic assessment

Preoperative Planning

Sonographic assessment, mainly through ultrasound and Doppler imaging, is valuable in preoperatively planning diverse medical conditions, ranging from vascular anomalies to skin lesions. Its diagnostic capabilities extend to precisely categorizing vascular anomalies and distinguishing between high- and low-flow lesions. This classification forms the foundation for subsequent workup, diagnosis, and management strategies [6]. Notably, the utility of preoperative ultrasound planning extends beyond vascular anomalies to include the surgical management of skin lesions, offering an efficient means of assessing both benign and malignant skin conditions [22]. Additionally, ultrasound plays a crucial role in the preoperative evaluation for vascular access planning [23]. Sonographic assessment significantly contributes to the comprehensive preoperative planning of various medical conditions, showcasing its versatility and importance in guiding clinical decisions and interventions.

Monitoring Treatment Response

Sonographic assessment demonstrates diverse clinical applications, notably in monitoring treatment response across various medical domains. Within vascular anomalies, ultrasound is a valuable tool for evaluating the response to biological treatment in patients grappling with moderate-to-severe plaque psoriasis [24]. Integrating dermoscopy and high-frequency ultrasound (HFUS) has proven promising in predicting and monitoring therapeutic response in this context [24]. In ocular oncology, ultrasound is crucial in assessing the treatment response of lesions such as uveal melanoma and retinoblastoma [25]. Through the measurement of thickness and the differentiation between flat and elevated lesions, ultrasound provides valuable insights for monitoring tumor growth and detecting local recurrence in treated uveal melanoma [25]. The clinical utility of sonography extends to the domain of vascular anomalies, where it is employed for both preoperative evaluation and monitoring treatment response [23]. This multifaceted application underscores the versatility of sonographic assessment, showcasing its significance in providing critical information for treatment planning and ongoing therapeutic management across a spectrum of medical conditions.

Guiding Interventions

Differentiation of high-flow and low-flow lesions: Sonography, in conjunction with Doppler imaging, is the primary imaging modality for the initial diagnosis of vascular anomalies. This approach enables the differentiation between high-flow and low-flow lesions based on the presence or absence of arterial flow within the lesion. Identifying arterial flow suggests a high-flow lesion, while the absence of arterial flow characterizes low-flow lesions [6]. This fundamental distinction is crucial for guiding subsequent diagnostic and management decisions.

Vessel density assessment: Upon detecting arterial flow within a lesion, a semiquantitative assessment of vessel density can be conducted using color Doppler imaging. This technique provides valuable insights into the vascular architecture of the lesion, contributing to a more comprehensive understanding of its hemodynamic characteristics [6]. This assessment aids in refining the characterization of vascular anomalies, contributing to a more nuanced diagnostic approach.

Treatment monitoring: Ultrasound's applicability extends to treatment monitoring, particularly in lesions such as uveal melanoma and retinoblastoma [25]. By utilizing ultrasound, healthcare professionals can assess the response of lesions to treatment, offering real-time insights into changes in size, characteristics, and vascularization. This monitoring aspect is critical in adapting and optimizing therapeutic strategies for improved patient outcomes [25].

Preoperative planning: Sonography is a valuable tool for preoperatively assessing benign and malignant skin lesions. This application enables healthcare providers to meticulously plan surgical interventions by accurately evaluating lesion characteristics and spatial relationships. The detailed insights gained through sonography enhance surgical precision and patient care [22].

Vascular access evaluation: In vascular access planning, ultrasound plays a pivotal role in the clinical evaluation of patients. It complements the initial history-taking and physical examination by providing detailed imaging of the vascular access site. Additionally, ultrasound guides the placement of catheters and other interventional devices, ensuring precision and minimizing procedural risks [23]. This application is particularly relevant in medical contexts where vascular access is crucial to patient care, such as in critical care or interventional procedures.

Challenges and limitations

Operator Dependence

The accuracy of ultrasound assessment is contingent upon the proficiency and experience of the operator. The potential for misdiagnosis or delayed diagnosis arises when ultrasound findings are inaccurately interpreted. Such inaccuracies can significantly influence the selection of treatment options and ultimately impact patient outcomes [8].

Limited Resolution

Ultrasound exhibits a resolution limitation compared to alternative imaging modalities like MRI and CT. This limitation poses challenges in visualizing small vessels and subtle changes in flow dynamics, often critical factors in diagnosing and managing slow-flow vascular malformations [8]. The inability to capture fine details may hinder the comprehensive evaluation of these conditions.

Artifacts

Ultrasound images are susceptible to artifacts, distortions, or anomalies caused by various factors such as tissue density, motion, or equipment settings. These artifacts can complicate the accurate assessment of flow and velocity characteristics in slow-flow vascular malformations, introducing potential pitfalls in interpretation [8]. Awareness of and mitigation strategies for artifacts are crucial for precise imaging.

Inability to Assess All Types of Vascular Malformations

While ultrasound is a valuable tool for evaluating slow-flow vascular malformations, it may only be universally suitable for some types. For instance, diagnostic arterial angiography is not indicated for slow-flow malformations, as they typically feature hypodynamic small arteriovenous fistulas [8]. The specificity of ultrasound's application underscores the need for a discerning approach in selecting imaging techniques.

Subtype-Specific Imaging Findings

The diagnostic efficacy of ultrasound in slow-flow vascular malformations hinges on the specific subtype of the malformation. Unique imaging characteristics associated with different subtypes can pose challenges in identification and interpretation. The subtype-specific nature of findings necessitates a nuanced understanding for accurate diagnosis [8].

Technical Challenges

Limited resolution: Ultrasound faces constraints in resolution, presenting challenges in visualizing small vessels and effectively distinguishing between slow-flow and high-flow lesions. The intricacies of vascular structures may be challenging to discern with the limited resolution of ultrasound [7].

Artifacts: The occurrence of artifacts, including reverberation and shadowing, during ultrasound imaging can impede the accurate assessment of slow-flow vascular malformations. These artifacts may introduce distortions that compromise the fidelity of the imaging data [7].

Inadequate imaging: Ultrasound imaging may prove insufficient in some instances, influenced by factors such as patient size, body habitus, or the location of the lesion. These variables may hinder the acquisition of a clear image of the slow-flow vascular malformation, limiting the diagnostic utility of ultrasound [7].

Inability to differentiate between slow-flow and high-flow lesions: While ultrasound can reveal slow blood flow within dysplastic vessels, its ability to consistently differentiate between slow-flow and high-flow lesions may be limited. This potential limitation poses a risk of misdiagnosis in distinguishing the hemodynamic characteristics of these vascular anomalies [7].

Limited sensitivity: Ultrasound may exhibit reduced sensitivity compared to alternative imaging modalities like MRI or CT in detecting slow-flow vascular malformations. Certain subtleties in pathology may be more effectively captured by these alternative methods [7].

User-dependent: The quality of ultrasound assessment is inherently user-dependent, influenced by the skill and experience of the sonographer. Variability in image interpretation may arise, emphasizing the importance of a skilled operator in ensuring accurate assessments of slow-flow vascular malformations [7].

Limitations in Specific Anatomical Locations

PSFVMs pose inherent challenges and limitations, particularly in specific anatomical locations. The diverse manifestations of PSFVMs are contingent upon their size and location, with anomalies affecting major central veins like the vena cavae, portal, iliac, and renal veins. VMs, for instance, have the potential to induce congestive pain and elevate the risk of thromboembolic events. In the gastrointestinal tract, these malformations may lead to bleeding, while lymphatic anomalies are susceptible to infection and leakage. Aberrations in central conducting lymphatic channels can give rise to complications such as chylothorax, chylous ascites, pulmonary dysfunction, chylous leaks through the skin, and bony destruction. Intra-articular vascular anomalies, though relatively rare, represent a subset of both high- and low-flow malformations, exhibiting diverse presentations with intra-articular and intraosseous extensions [4,13]. Low-flow vascular malformations are predisposed to stasis and intraluminal clotting due to their inherently low-flow nature. In such instances, exuberant intralesional intraluminal capillary ingrowth, mimicking high-flow lesions, may be observed on imaging [16]. A linear high-frequency transducer (12-17 MHz) is optimal for ultrasound examination of vascular malformations, although more profound malformations may necessitate lower frequencies. Ultrasound's effectiveness can be constrained in specific anatomical regions, such as the skull base, spine, and deep pelvic structures, where bone and gas hinder visualization. In these scenarios, the utilization of MRI or CT may become imperative [7].

Comparative analysis with other imaging modalities

MRI

Incorporating MRI in the comparative analysis of imaging modalities is imperative for comprehensively evaluating PSFVMs. Post-contrast MRI is a valuable tool for scrutinizing the venous system's drainage extent and characterizing slow-flow malformations, particularly those linked to additional anomalies. MRI/angiography is crucial in defining the morphology of high-flow lesions like AVMs. Furthermore, established MRI techniques, including magnetic resonance venography (MRV), contribute significantly to diagnosing slow-flow vascular malformations, offering intricate insights into flow characteristics and anatomical extension. MRI's utility extends to mapping the vascular supply of high-flow lesions, enhancing the initial sonographic assessment. MRI is indispensable for diagnosing and classifying PVMs, providing detailed information on flow dynamics and anatomical features [4,8]. The well-established diagnostic value of MRI in assessing slow-flow vascular malformations complements the insights gained from ultrasound and Doppler imaging. While ultrasound, especially color Doppler and spectral analysis, is adept at confirming high-systolic flow in high-flow lesions, MRI offers detailed morphological information. It proves essential for characterizing slow-flow malformations, particularly in elucidating their anatomical extension and flow patterns [4,6]. MRI is an indispensable imaging modality, contributing significantly to the comprehensive assessment of PSFVMs. It provides invaluable details on anatomical morphology, flow dynamics, and anomaly characterization, complementing the initial sonographic evaluation.

CT

The sonographic assessment of PSFVMs is pivotal in the diagnostic process, significantly classifying these anomalies. Paired with Doppler imaging, sonography is the primary imaging modality for diagnosing vascular anomalies, particularly in pediatric cases. Its ability to discern between high- and low-flow lesions, encompassing capillary, venous, and lymphatic malformations, is crucial to the diagnostic journey. When integrated with clinical findings, sonography facilitates accurate clinic radiological diagnoses in most cases. While further imaging through MRI or CT is commonly employed, especially for high-flow lesions, sonography's value lies in differentiating vascular and lymphatic malformations and confirming high-systolic flow in high-flow lesions. Sonography is indispensable in the holistic evaluation and diagnosis of PSFVMs, particularly in pediatric populations [6,8]. Various imaging modalities' diagnostic landscapes, including ultrasound, MRI, and CT, have undergone extensive scrutiny concerning different vascular malformations. Ultrasound's significance lies in its role in initial assessments and categorizing vascular anomalies, whereas MRI proves instrumental in evaluating venous system drainage extent and characterizing slow-flow malformations. Conversely, CTA excels in mapping the vascular supply of high-flow lesions [6,8]. While sonography takes the lead in the preliminary assessment of PSFVMs, MRI and CT complementarily contribute to further characterizing these anomalies. Their roles become incredibly prominent in distinguishing between high- and low-flow lesions and mapping the vascular supply of high-flow lesions [6,8].

Angiography

In the diagnostic realm of vascular malformations, imaging modalities such as angiography, MRI, and CT are frequently employed to complement sonographic assessments. Angiography, although invasive, stands out for selectively assessing arteriovenous shunting, nidus, and feeding vessels in fast-flow malformations [8]. Meanwhile, MRI and MR angiography are crucial in mapping the vascular supply of high-flow lesions and distinguishing between high- and low-flow lesions [17,26]. Dynamic MRI has proven efficacy in discerning between high-flow and low-flow vascular malformations [17]. CT angiography and 4D CTA also find application in evaluating vascular malformations [8]. Despite the versatility of these advanced imaging modalities, sonography, often coupled with clinical findings, retains its status as the primary, non-invasive imaging modality for the initial diagnosis of vascular anomalies, especially in the pediatric population [6,8].

Future directions and research opportunities

Advancements in Sonographic Technology

Recent years have witnessed notable trends in sonographic technology, driven by advancements that aim to enhance reproducibility and minimize user interaction through automation and AI integration [10]. Leading ultrasound systems like the Philips Epiq and Affiniti are at the forefront, incorporating anatomical intelligence that facilitates visual mapping and annotation of screened anatomy with minimal user intervention. Concurrently, the rise of portable ultrasound devices tethered to smartphones or tablets has gained traction, featuring specialized applications tailored for cardiac and vascular imaging, among others [10]. These portable devices offer quantification and functionalities traditionally associated with larger cart-based systems. The evolution of molecular ultrasound imaging emerges as a promising frontier, poised to deliver high spatial and temporal resolution, real-time imaging, and non-invasiveness for detecting molecular targets [27]. Ongoing developments focus on the clinical translatability of molecular ultrasound contrast microbubbles, expanding the potential clinical applications of this innovative technology. In the specific context of evaluating PSFVM, the recommendation leans towards using high-frequency linear transducers (12-17 MHz) for optimal imaging [7]. In summary, the continual progress in sonographic technology is enhancing the precision and efficiency of diagnostic imaging, with exciting prospects shaping the future of this field [7,27-29].

Standardization of Sonographic Criteria for PSFVM

Developing a standardized set of sonographic criteria for PSFVMs is a crucial step in enhancing the reproducibility and accuracy of diagnoses. Collaborative efforts involving sonographers, radiologists, and other medical professionals are essential to establish a consensus on the most relevant and reliable sonographic features for diagnosing PSFVMs [6,28]. This standardized approach ensures consistency across assessments and facilitates more reliable diagnoses.

Integration of AI and automation: Leveraging AI and automation can significantly enhance the reproducibility of sonographic assessments, irrespective of the sonographer's experience level. Developing advanced ultrasound systems with anatomical intelligence, visual mapping, and annotation capabilities minimizes user interaction, providing a more standardized and reliable diagnostic process [28]. This integration of AI contributes to more consistent and accurate results in the sonographic evaluation of PSFVM.

Molecular ultrasound imaging: The exploration of 3D approaches for molecular ultrasound imaging in vivo represents a promising avenue for improving diagnostic capabilities in sonography. Ongoing developments in clinically translatable molecular ultrasound contrast microbubbles aim to broaden the applications of molecular ultrasound imaging, potentially offering enhanced insights into the characteristics of PSFVMs [27]. This innovative approach opens new possibilities for non-invasive and detailed assessments.

Multimodal imaging: Integrating sonography with other imaging modalities, such as MRI or CT, provides a more comprehensive evaluation of PSFVM. This multimodal approach enhances diagnostic accuracy by offering complementary information from different imaging techniques [6]. Combining sonography with other modalities contributes to a more thorough understanding of vascular anomalies, aiding in precise diagnosis and management.

Pediatric-specific research: Directing research efforts toward the sonographic assessment of PSFVMs in the pediatric population is essential for gaining insights into specific ultrasound characteristics and diagnostic challenges in children. This focused approach contributes to refining the accuracy of sonographic assessments and improves the overall management of PSFVMs in pediatric patients [6]. Pediatric-specific research ensures that diagnostic practices are tailored to the unique aspects of vascular anomalies in children, optimizing patient care.

Multi-Modal Imaging Approaches

The future trajectory and research prospects in multi-modal imaging, specifically within neuroimaging, encompass the heightened integration of "neurochemical" imaging modalities, such as MR-spectroscopy, with structural and functional modalities. This amalgamation promises to yield a more exhaustive comprehension of brain function and connectivity. Moreover, the field stands to gain significantly from the judicious application of multimodal approaches in clinical and translational research, necessitating open science and improved accessibility of tools for processing and analyzing multimodal data [30]. Multimodal neuroimaging, which amalgamates information from various imaging modalities, is a primary driver in current research across neuroscience, neurology, psychiatry, and neurosurgery. It affords a more holistic depiction of brain structure and function by surmounting the constraints of individual modalities. Challenges persist in fully grasping the intercorrelation between different modalities, yet the potential to pinpoint disease biomarkers with heightened specificity and sensitivity opens new avenues for advancing brain research. The imminent proliferation of novel neuroimaging scanners, particularly hybrid ones, is poised to propel the field of multimodal neuroimaging research further [31]. Within molecular imaging, the emergence of multimodal molecular imaging, which harmonizes two or more imaging modalities to leverage their strengths and offset their limitations, represents an interdisciplinary methodology with profound implications for disease diagnosis, treatment, and drug development. The future trajectory in this realm entails the ongoing refinement and integration of diverse imaging modalities to elevate the precision and reliability of disease imaging and diagnosis [31].

## Conclusions

In conclusion, the review of the sonographic assessment of PSFVMs underscores the significant diagnostic capabilities of this imaging modality. The key findings reveal that sonography exhibits high diagnostic accuracy in identifying morphological and hemodynamic features critical for accurately classifying PSFVMs and distinguishing them from other vascular anomalies. The non-invasive nature and real-time imaging capabilities make sonography a practical and cost-effective tool for early diagnosis and preoperative planning, ultimately guiding interventions and improving patient outcomes. However, the review also highlights limitations, such as operator dependence and challenges in visualizing lesions in specific anatomical locations, emphasizing careful interpretation. The implications for clinical practice emphasize the pivotal role of sonography in early identification and intervention for PSFVMs, recommending its integration into multimodal approaches for a comprehensive understanding. Ongoing education and training are crucial to ensuring proficiency among healthcare professionals. Recommendations for future research include the standardization of sonographic criteria, conducting longitudinal studies to assess treatment response, and comparative studies with other imaging modalities to refine the role of sonography in the management of PSFVMs. Addressing these aspects will contribute to the continued advancement of sonographic techniques in the comprehensive assessment and treatment of PSFVMs.
